# IS ONE MONTH OF EXERCISE-BASED CARDIAC REHABILITATION SUFFICIENT AFTER SURGICAL AORTIC VALVE REPLACEMENT? A FEASIBILITY STUDY OF A RANDOMIZED CONTROLLED TRIAL

**DOI:** 10.2340/jrm-cc.v8.43087

**Published:** 2025-09-30

**Authors:** Malin Schlyter, Eva Ekvall Hansson

**Affiliations:** 1 Department of Cardio thoracic surgery, Skåne University Hospital, Lund, Sweden; 2 Human Movement: Health and Rehabilitation Research Unit, Department of Health Sciences, Lund University, Lund, Sweden

**Keywords:** aortic stenosis, surgical aortic valve replacement, physical activity, functional exercise capacity, exercise-based cardiac rehabilitation

## Abstract

**Objective:**

To study whether there were differences in terms of change in the level of physical activity and functional exercise capacity between 1 month and 3 months of training after surgical aortic valve replacement.

**Design:**

A feasibility study of a randomized controlled trial.

**Subjects:**

After drop-out, a total of 30 patients with aortic stenosis participated in the 2 interventions.

**Methods:**

Group A received 1 month and group B 3 months of cardiac rehabilitation after surgical aortic valve replacement. Feasibility was measured in terms of recruitment, adherence and retention rate, adverse events and the ability to collect primary and secondary outcome measurements.

**Results:**

Regarding feasibility, the recruitment rate was low (55%), but the adherence and retention rates were good (group A 81%/94%, group B 64%/79%). The outcome assessment collection was good, and there was only 1 adverse event. Significant differences were found regarding physical activity and self-perceived health in favour of group B.

**Conclusion:**

This feasibility study showed that although the recruitment rate was low, other measures were satisfactory. Results indicate that a shorter supervised programme may be sufficient and possibly facilitate more effective use of resources.

## LAY ABSTRACT

To study whether there were differences in terms of change in the level of physical activity and functional exercise capacity between 1 month and 3 months of training after open heart surgery due to aortic stenosis.

Thirty-six patients with a mean age of 71.4 years were included in this study. After drop-out, 30 patients participated in the 2 interventions. Group A received 1 month and group B 3 months of cardiac rehabilitation after open heart surgery due to aortic stenosis.

Significant differences were found regarding physical activity and self-perceived health in favour of group B.

Results indicate that a shorter supervised programme may be sufficient and possibly facilitate more effective use of resources.

Aortic stenosis (AS) is a common disease amongst the older population, and the number of patients is expected to increase (1, 2). After hypertension and coronary artery disease (CAD), AS is the most common cardiac disease in developed countries (3). It is characterized by narrowing of the aortic valve due to calcification and stiffness of the cusps and surrounding structure, resulting in increasing strain on the left ventricle and haemostatic effects. Heart valve diseases may result in valvular dysfunction and heart failure (4).

AS prognosis is markedly impaired after the occurrence of symptoms, the most common being dyspnoea, fatigue, angina pectoris, syncope or near syncope (4).

Surgery should be considered in symptomatic persons with AS and in asymptomatic persons when they exhibit an abnormal response to exercise or have moderate to severe calcification of the aortic valve or left ventricular dysfunction (ejection fraction [EF] < 50%) (3). In the case of concomitant obstructive CAD, the patient can be revascularized during surgical aortic valve replacement (sAVR) (1).

Many patients on the waiting list for surgery minimize their physical activity, resulting in anxiety, reduced physical and social functions, and poorer vitality and general health (5). The disease can also result in kinesiophobia (fear of movement), less activity in daily life and reduced physical fitness (2). Kinesiophobia has been shown to decrease when the person attends cardiac rehabilitation (CR) after a cardiac event (6). The guidelines from the National Board of Health and Welfare underline the importance of offering exercise-based training in the form of CR to this group of patients (2). There is a minimal risk of significant adverse events (1, 7, 8) with CR, which improves morbidity, quality of life (QoL) and exercise capacity (7, 9).

Patients are more likely to be referred to CR after combined heart valve surgery and coronary artery bypass grafting (CABG) because the CR guidelines for heart valve surgery patients are based on evidence from patients with ischaemic heart disease (10). CR and physical activity are recommended treatments after heart valve surgery (10).

The recommendation from The National Board of Health and Welfare (2) is 3 months of CR after cardiac surgery. However, it is uncertain how well the recommendations are followed. In patients with CAD, it has been shown that 3 months of CR is the gold standard. Few studies concerning CR have been conducted on patients after sAVR (9); thus, it is unknown if 1 month of CR might be sufficient.

The aim was to study the feasibility of a randomized controlled trial (RCT) investigating whether there were any differences in the level of physical activity and functional exercise capacity after 1 month of training after isolated sAVR or sAVR combined with CABG compared to “the gold standard”, that is 3 months of training.

## METHODS

### Study design

A feasibility study of a randomized controlled trial with 1 group receiving 1 month of CR (group A) after isolated sAVR or sAVR combined with CABG versus 1 group receiving 3 months of CR (group B).

### Selection

Persons on the waiting list at Skåne University Hospital in Lund, Sweden, for isolated sAVR or sAVR combined with CABG with an EF of > 30% and living in the Lund catchment area were asked to participate in the study by means of written information included in the appointment for surgery (randomized convenience sample). The patients were then contacted by phone after 1 week and given further information and an opportunity to ask questions. The participants should be able to understand spoken and written Swedish, and those with known cognitive difficulties were excluded. Despite not being included in the study, these 2 groups were still offered the opportunity to take part in CR.

**Fig.1:**
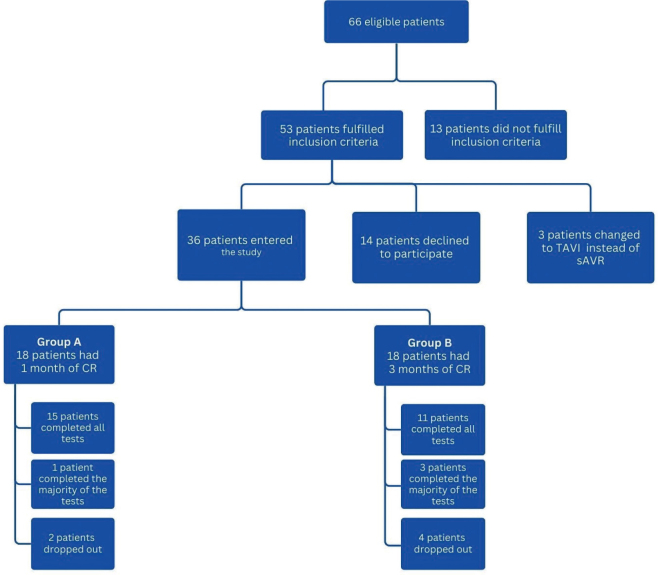
Flow chart of the study.

Patients were enrolled in the study from September 2017 to May 2019. Of the 66 eligible patients, 30 agreed to participate in this study (Fig. 1).

### Ethical considerations

It was important to point out that participation was voluntary, and that the participants could withdraw from the study at any time. The Regional Ethical Review Board in Lund approved this study in June 2017 (Approval number 2017/422).

### Settings

This study was conducted at Skåne University Hospital in Lund, which has 1 of 8 thoracic surgery clinics that perform cardiac surgery in Sweden.

### Intervention

The interventions comprised 1 month of training twice per week or 3 months of training twice per week. Each training session started with warming up on a cycle ergometer, followed by circuit training focusing on increased aerobic capacity and muscle strength. The training session ended with relaxation. Warming up and cooling down as well as relaxation techniques are recommended in CR after heart valve surgery (11). Blood pressure and pulse were monitored, and the patients were asked for their perceived rating of exertion in accordance with the Borg Scale (12).

### Procedure

The patients were offered CR twice per week for 1 month (group A) or 3 months (group B). After agreeing to participate, the participants did their first test and were randomized to group A or group B. Sealed envelopes were randomly drawn from a box by an independent person who was not involved in this study. The participants received the information about which group they had drawn the day after surgery.

### Measures

The participants were tested 5 times: the day before surgery, before they started exercise-based CR (4–6 weeks after surgery), after 1 month of exercise-based CR, after 3 months of exercise-based CR and 6 months after starting exercise-based CR regarding the following feasibility outcome measures:

*Recruitment rate* – how many patients who met the inclusion criteria agreed to participate (%)?

*Adherence rate* – how many training sessions did the participants take part in (%)?

*Retention rate* – how many patients completed the follow-up tests (%)?

### Adverse events

*Ability to collect primary and secondary outcome measurements* at all assessment points. What percentage of possible outcomes were actually collected?

### Primary outcome measure for functional exercise capacity

*6MWT.* Walking distance was measured with the 6-min walk test (6MWT) (13–15) conducted indoors along a flat, straight 25 m long corridor. The participants were instructed to walk as far as possible for 6 min. They were permitted to slow down, stop and rest if necessary but resume walking as soon as possible. Blood pressure, heart rate and SpO_2_ were measured before the test, whilst heart rate, SpO_2_ and rating of perceived exertion and dyspnoea were measured after the test.

There are several exercise tests for evaluating functional capacity. The 6MWT is easy to administer and perform, better tolerated and more reflective of activities of daily living than other walking tests (14, 15).

The most common indication for the 6MWT is the need to measure the response to medical interventions in patients with moderate to severe heart or lung disease. It can also be used before and after an intervention such as exercise-based CR or to measure functional status as a predictor of morbidity and mortality (14).

Because encouragement can affect test results, in the present study, pre-decided sentences of encouragement were used with all patients at specific times during the tests (14–16). Blood pressure, heart rate and oxygen saturation were noted as well as dyspnoea and overall fatigue using the Borg scale. The total distance walked was calculated, rounded to the nearest metre (14).

The 6MWT was chosen as a safe and feasible test for patients with AS because the cardiopulmonary exercise test (CPET) is contraindicated for patients with AS before surgery (17). However, CPET is commonly used before CABG to evaluate functional capacity (13).

### Secondary outcome measures

*Heel-lift.* To assess the dynamic endurance of the calf muscle, the participant performed a maximal unilateral heel-lift on a 10-degree tilted wedge, 1 lift every other second using a metronome (Seiko SQ60 Metronome). The participant touched the wall with the fingertips to maintain balance, and the contralateral foot was held slightly above the floor. The number of maximal heel-lifts was counted (18).

### 30-second chair stand test

One of the most common activities of daily living is rising from a seated to a standing position. The ability to perform a sit-to-stand movement is essential for maintaining physical independence. It may be one of the most important functional measures of physical capacity (19) and is both quick and reliable (20). Chair stand tasks also require posture control, joint flexibility, cardiovascular capacity (21), balance, sensorimotor and psychological factors (19).

The participants were asked to make as many full stands as possible from a seated position (stand up from and sit down on a chair), without using their arms during a 30-second period (20, 21). The score was the number of full stands completed in 30 s (21).

*Self-perceived physical capacity* was measured by a 100 mm VAS (22). The participants were asked to rate their self-perceived physical capacity from 0 (worst possible physical capacity) to 100 (best possible physical capacity) at all assessment points.

*The level of physical activity* was measured with questions concerning physical activity and physical training in accordance with Haskell et al. (23), and by using Frändin and Grimby’s activity scale (24). We chose to evaluate the level of physical activity with the 2 above-mentioned methods as they have already been tested and used on patients in CR after cardiac surgery (CABG). Haskell (23) investigates the level of physical activity and the level of exercise during the past week on an 8 grade scale. Frändin and Grimby (24) ask about the level of physical activity during the past week on a 6 grade scale.

We evaluated which *factors limit functional exercise capacity in daily life*: chest pain, dizziness, dyspnoea, leg fatigue, general fatigue, fear of movement, pain (other than chest pain), other disabilities, dissuaded by relatives/healthcare professionals to exert physical effort (22). The current state of self-perceived health was measured by the *EuroQol 5-dimensions Visual Analogue Scale (EQ5D VAS)*, in which the participants were asked to rate how good or bad their overall health was at all assessment points, 0 being the worst possible health and 100 being the best possible health.

These tests are designed and used for patients after myocardial infarction and CABG but can be and are also used after sAVR/sAVR and CABG. The tests are registered in SEPHIA (secondary prevention after care at a heart intensive ward) (22), a quality register for patients after myocardial infarction and in some places after CABG.

### Statistical analysis

Based on previous studies in which the 6MWT was used, with a clinically significant difference in walking distance of 54 metres and a standard deviation of 56 metres, 38 participants were needed in each group to reach a power of 80% at a significance level of 0.05 (25, 26). Continuous, normally distributed variables were expressed as means and standard deviations. Categorical variables were presented as frequencies and percentages. Non-normally distributed continuous variables were expressed as medians and interquartile ranges. For comparison between groups, the Mann-Whitney U 2 independent samples test was applied. All statistical analyses were performed using IBM SPSS Statistics version 25.

## RESULTS

### Participants

Sixty-six patients were eligible for the study. Thirteen did not fulfil inclusion criteria. Of the remaining 53 patients, 14 declined to participate and 3 had their surgery changed from sAVR to transcatheter aortic valve implantation (TAVI), resulting in 36 patients entering the study. A total of 6 patients dropped out during the study due to various medical conditions, resulting in 30 patients participating in the 2 interventions (Fig. 1). The total study sample comprised 12 women and 24 men, with a mean age of 71.4 years. 77.8% had isolated sAVR and 75% were retired. 69.4% had 1–3 risk factors for cardiovascular disease. The 2 groups were similar at baseline except for gender. The gender distribution in group A is what is normally seen in the population undergoing cardiac surgery (Table I).

**Table I:** Baseline characteristics of the patients in the study

		All	Group A (1 month of CR)	Group B (3 months of CR)
**Gender** F/M	*n* (%)	12/24 (33/67)	4/14 (22/78)	8/10 (44/56)
**Age** (years)	mean (SD)	71.4 (7.4)	69.9 (6.7)	72.9 (7.9)
**BMI**	mean (SD)	27.6 (4.0)	28.0 (4.6)	27.2 (3.3)
**EF** normal/decreased	*n* (%)	30/6 (83/17)	16/2 (89/11)	14/4 (78/22)
**Surgery** sAVR/sAVR combined with CABG	*n* (%)	28/8 (78/22)	14/4 (78/22)	14/4 (78/22)
**Education** University/Upper secondary school/Elementary school	*n* (%)	9/11/16 (25/31/44)	4/5/9 (22/28/50)	5/6/7 (28/33/39)
**Civil status** Married/partner/Living apart/Living alone	*n* (%)	20/3/13 (56/8/36)	8/1/9 (44/6/50)	12/2/4 (67/11/22)
**Smoking habits** Never smoked/Former smoker/Smoker	*n* (%)	18/17/1 (50/47/3)	9/9/0 (50/50)	9/8/1 (50/44/6)
**Occupation** Retired/working	*n* (%)	27/9 (75/25)	13/5 (72/28)	14/4 (78/22)
**Risk factors**				
0	*n* (%)	6 (17)	2 (11)	4 (22)
1		9 (25)	5 (28)	4 (22)
2		7 (19)	3 (17)	4 (22)
3		9 (25)	5 (28)	4 (22)
4		2 (6)	2 (11)	0
5		2 (6)	1 (6)	1 (6)
6		1 (3)	0	1 (6)

BMI: body mass index; EF: ejection fraction; sAVR: surgical aortic valve replacement; CABG: coronary artery bypass grafting; CR: cardiac rehabilitation.

Risk factors: coronary artery disease (CAD), previous myocardial infarction (MI), previous percutaneous coronary intervention (PCI), previous CABG, hypertension, atrial fibrillation (AF), diabetes, previous stroke/TIA, kidney disease, pulmonary disease, peripheral vascular disease, other valve disease, hyperlipidaemia, heredity, heart failure, overweight.

Results did not differ between the 2 groups at baseline (Table II). Only one participant used a walking aid (a crutch) at the postoperative test before training because of dizziness on that day.

**Table II:** Baseline results of the patients in this study

		All	Group A (1 month of CR)	Group B (3 months of CR)
**Walking distance** (meter)	mean (SD)	425.2 (80.5)	435.3 (88.6)	415.1 (72.7)
**Dyspnoea** (CR-10)	median (IQR)	3.0 (1.0–3.0)	2.5 (1.0–3.0)	3.0 (1.0–3.0)
**Rating of perceived exertion** (Borg scale)	median (IQR)	13.0 (11.0–13.0)	12.5 (9.0–13.2)	13.0 (11.8–13.2)
**Chair stand test**	mean (SD)	11.4 (5.1)	11.1 (6.0)	11.6 (4.2)
**Heel lift**	mean (SD)	15.6 (7.4)	14.8 (6.5)	16.3 (8.3)
**Physical capacity** (0–100)	mean (SD)	53.4 (23.6)	53.6 (26.8)	53.3 (20.8)
**Haskell I (mild dyspnoea)** (0–7 days)	median (IQR)	2.0 (1.0–7.0)	2.5 (1.0–7.0)	2.0 (0.8–6.2)
**Haskell II (severe dyspnoea)** (0–7 days)	median (IQR)	0 (0–2.0)	0 (0–2.0)	0.5 (0–2.0)
**Frändin and Grimby activity scale** (level 1–6)	median (IQR)	3.0 (3.0–3.0)	3.0 (2.8–3.2)	3.0 (3.0–3.0)
**Limited everyday life** no/yes	*n* (%)	9/27 (25/75)	4/14 (22/78)	5/13 (28/72)
**Number of limiting factors** 0/1/2	*n* (%)	9/18/9 (25/50/25)	4/10/4 (22/56/22)	5/8/5 (28/44/28)
**Limiting factors**	Most common	Dyspnoea, chest pain	Dyspnoea, fatigue	Dyspnoea, chest pain
**EQ5D VAS** (0–100)	Mean (SD)	62.7 (21.9)	63.6 (23.3)	61.7 (21.0)

CR: cardiac rehabilitation.

### Feasibility measures

*Recruitment rate* – 55%: Of the 66 patients who met the inclusion criteria, 36 agreed to participate.

*Adherence rate* – In the group training for 1 month (group A), 81% (13 out of 16 patients) participated in all the training sessions, and in the group training for 3 months (group B), the corresponding number was 64% (9 out of 14 patients).

*Retention rate* – In group A, 94% (15 out of 16 patients) completed all follow-up tests, and in group B, the corresponding number was 79% (11 out of 14 patients). See flow chart for participants and dropouts (Fig. 1).

Of the dropouts in group A (2 patients), 1 dropped out after the preoperative test and the other after the postoperative test and 2 training sessions because of dizziness. Of the dropouts in group B (4 patients), 3 patients dropped out after the preoperative test and 1 patient after the postoperative test but before the start of CR, all due to medical reasons.

*Adverse events*: there was 1 adverse event when a patient fainted at the end of the first training session.

*The ability to collect outcome measurements* preoperatively was 100%. Postoperatively before training: 86%, after 1 month of training: 83%, at 3 months from the start of training: 81% and at 6 months from the start of training: 72%.

### Outcome measures

The Frändin and Grimby activity scale (24) revealed no difference in the level of physical activity between the 2 groups. Concerning physical activity and physical training based on Haskell et al. (23), there was a difference at Haskell I (mild dyspnoea) after 1 month (*p* = 0.007) and after 6 months (*p* = 0.03) in favour of group B (Table III).

**Table III:** Differences between the groups in terms of change before and after training in exercise-based cardiac rehabilitation

	Change after compared with before (median, CI)								
Outcomes	Difference in terms of change between the groups 1 month after training compared with before training		*p*	Difference in terms of change between the groups after 3 months compared with before training		*p*	Difference in terms of change between groups after 6 months compared with before training		*p*
	Group A *n* = 16	Group B *n* = 14		Group A *n* = 15	Group B *n* = 14		Group A *n* = 15	Group B *n* = 11	
**6 min walking distance (meter)**	26.5 (16–50)	26.5 (6–47)	0.119	41.0 (4–74)	70.0 (34–101)	0.220	55.0 (31–104)	60.0 (31–73)	0.516
**Chair stand test (times)**	2.0 (1–3)	3.0 (1–3)	0.457	2.0 (1–3)	4.5 (2–7)	0.089	2.0 (1–4)	4.0 (1–5)	0.476
**Heel lift (times)**	3.0 (-3–7)	1.0 (0–7)	0.504	1.0 (-2–8)	4.0 (1–10)	0.304	1.0 (-2–7)	3.0 (0–8)	0.348
**Physical capacity (0–100)**	8.0 (2–5)	5.0 (-10–40)	0.967	5.0 (-3–29)	16.5 (2–48)	0.275	19.0 (4–35)	17.0 (7–37)	0.608
**Haskell I (0–7)**	0.0 (-1–2)	-2.0 (-5–0)	**0.007**	0.0 (-2–2)	-0.5 (-3–0)	0.246	0.0 (-2–3)	-2.0 (-7–0)	**0.030**
**Haskell II (0–7)**	0.0 (0–2)	1.0 (0–2)	0.859	1.0 (0–3)	2.0 (0–3)	0.610	0.0 (-1–1)	0.0 (0–2)	0.182
**Frändin & Grimby (1–6)**	1.0 (1–1)	0.0 (0–1)	0.290	1.0 (1–1)	1.0 (0–2)	0.154	1.0 (1–1)	1.0 (0–1)	0.816
**EQ5D VAS (0–100)**	0.0 (-11–10)	10.5 (0–21)	0.055	0.0 (-7–13)	10.0 (1–36)	**0.047**	5.0 (-5–26)	10.0 (0–50)	0.221

Median, 95% (Group A: 1 month of CR, Group B: 3 months of CR) CI and *p*-value**After surgery, but before and after training. *P*-values in bold indicate a significant difference between the 2 groups.

No significant difference was found between the 2 groups in functional exercise capacity measured with the 6MWT, CST or heel-lift.

At the 3-month follow-up, group B’s (training for 3 months) 6-min walking distance (6MWD) increased more than that of group A (training for 1 month), but the increase was not statistically significant. This difference was not maintained at the follow-up 6 months after surgery (Table III).

The current state of self-perceived health measured with the EQ5D VAS showed a significant difference between the 2 groups after 3 months (*p* = 0.047) in favour of group B (Table III).

Before surgery, the most common factors that limited functional exercise capacity in daily life were dyspnoea and chest pain. After surgery but before the start of CR, limiting factors were general fatigue and dyspnoea, after 1 month in CR other pain (not chest pain) and dizziness, at 3 months dyspnoea and leg fatigue and at 6 months dizziness and chest pain.

## DISCUSSION

### Summary

In this feasibility study of a randomized controlled trial, the recruitment rate was average. The adherence and retention rate was high in the group that had trained for 1 month and a little lower in the group that had trained for 3 months.

Regarding change in the level of physical activity or functional exercise capacity, there was no significant difference between those who had 1 versus 3 months of exercise-based CR either at baseline, before training or after 1, 3 and 6 months.

### Discussion of results

In this study, both groups increased their 6MWD by an average of 59 metres, which, despite the small sample, is a clinically relevant improvement (25, 26). In view of the fact that there was no significant difference between the 2 groups in our study, one could discuss the possibility of offering at least 1 month of exercise-based CR to all patients who suffer a cardiac event. This is in line with another study about pulmonary rehabilitation for 4 versus 7 weeks (27), where there were no significant differences between the groups after 7 weeks or at follow-up after 6 months. The results in our study indicate that it did not make a significant difference whether the patients attended exercise-based CR for 1 or 3 months in terms of remaining active at the 6-month follow-up. Those who had an active lifestyle before surgery continued being active. These results indicate that a shorter supervised programme may facilitate more effective use of resources, possibly resulting in making rehabilitation available to more patients (27).

After a cardiac event, patients need structural support in the shape of exercise-based CR to maintain or improve functional capacity (28). Despite the fact that CR is a cost-effective intervention, it is underused (13, 28). Barriers to participation in CR include lack of physician involvement, lack of self-motivation, depression and anxiety, lack of familial support, lack of time, transport difficulties and dislike of group settings (29). Patients with a positive approach towards their recovery tend to overcome the challenge posed by their cardiac event and were found to exercise more (29). Benefits of CR include a safe place to exercise, improved self-confidence, as well as staff and peer group support. However, we have not had the opportunity to investigate these factors in the present study.

AS is sometimes caused by a congenital bicuspid aortic valve (BAV) (4). In this study, 9 of the 30 patients had a BAV, 3 women and 6 men. Those with a BAV may experience symptoms, thus require surgery earlier than patients with a tricuspid valve (30–32). This was also the case in our study, where the patients with BAV were somewhat younger.

### Strengths and limitations

Lund is 1 of 8 thoracic surgery clinics that perform cardiac surgery in Sweden. Patients from the southern part of Sweden who require cardiac surgery are treated there. All the follow-up tests were performed in Lund to ensure that they were carried out in an identical manner. As a result, the study only included patients living in a specific catchment area close to Lund. This limited the number of participants and might explain the low recruitment rate, which is the main limitation in this study as only 36 out of the 66 eligible patients agreed to participate (Fig. 1).

The examiner was the same physiotherapist who performed the randomization to the 2 groups and who conducted all the tests and analyses. Those participants attending exercise-based CR in Lund also met the same physiotherapist there. Although this led to a risk of bias, it was necessary for practical reasons.

Our study population is similar to that in other studies of patients with sAVR; the majority are men, and the average age is around 70 years (26, 33, 34). This indicates that the generalizability of a future larger RCT is probably good.

The tests were chosen because they are used in CR after myocardial infarction and CABG. The heel-lift test seemed to be hard to perform. Many of the participants had difficulties keeping pace despite the metronome and almost all slipped on the tilted wedge. The 6MWT and the CST were easy to perform and evaluate.

The level of physical activity was measured by questions concerning physical activity and physical training presented by Haskell et al. (23), who count the number of days that activation resulted in mild or severe dyspnoea. This test seemed to be difficult to interpret, as many participants stop exercising before the onset of dyspnoea. One can, nevertheless, be active, and although some participants become tired, they do not experience dyspnoea.

### Implications for future studies

This study indicates that 1 month of exercise-based CR might be sufficient after a cardiac event, in which case more patients could be offered CR with the same resources. Since this study was conducted, all patients are offered exercise-based CR after sAVR at Skåne University Hospital in Lund.

Before performing a full RCT, we recommend the following adjustments:

- Do not include the heel-lift test (18) because it was difficult for most of the participants to perform
- Do not use the Haskell questionnaire (23) as it was difficult to interpret

### Conclusion

This feasibility study of an RCT showed that the recruitment rate was low but other measures of feasibility were excellent. There were no differences in the 6MWT, heel-lift, CST or physical capacity between the groups. Both groups increased their 6MWD by an average of 59 metres, which is a clinically relevant improvement. In addition, significant differences were found regarding self-reported physical activity and self-perceived health in analyses of the 3-month training group. However, it seems that a shorter supervised programme might be sufficient and may facilitate more effective use of resources, resulting in exercise-based CR being offered to more patients after all cardiac events. This should be done to avoid kinesiophobia and to increase QoL after sAVR or sAVR combined with CABG.
